# Residential Mobility and Housing Tenure Among Immigrants and Their Descendants: a Cross-National Analysis of Five European Countries

**DOI:** 10.1007/s10680-025-09757-3

**Published:** 2025-11-24

**Authors:** Joseph Harrison, Isaure Delaporte, Hill Kulu, Júlia Mikolai, Chia Liu, Mary Abed Al Ahad, Julie Lacroix, Gunnar Andersson, Ariane Pailhé

**Affiliations:** 1 University of St Andrews, St Andrews, Scotland; 2 University of Salford, Salford, UK; 3 International Labour Organisation, Geneva, Switzerland; 4 Université de Neuchâtel, Neuchâtel, Switzerland; 5 Stockholm University, Stockholm, Sweden; 6 Institut National d’études Démographiques (INED), Paris, France

**Keywords:** Residential mobility, Housing tenure, Immigrants, Descendants of immigrants, Event history analysis, Cross-national comparison

## Abstract

Understanding the housing and residential mobility of migrant populations is crucial to facilitate their integration into host societies. Yet, little is known about their experiences across generations, origin groups, and country contexts. This study investigates residential mobility and housing changes among immigrants and their descendants in five European countries (United Kingdom, France, Germany, Switzerland, and Sweden) with different housing markets and migrant populations. Using longitudinal data from 2010 to 2019, we first compare the risk of a residential move across migrant generations, origins groups, and host countries. We then study the propensity to move to different housing tenure types (i.e., homeownership, private renting, and social renting). We find distinct patterns of residential moves among migrant generations and origin groups. First, immigrants’ residential mobility levels vary across origin groups and country contexts. However, we generally find lower mobility for the second generation compared to the first in all groups. Second, in all countries, immigrants, especially from non-European countries, are less likely to move to homeownership and more likely to move to social or private renting than the native populations. Some of the differences decline across migrant generations, however, we still find lower levels of mobility to homeownership and a higher propensity of moving to social renting among some descendant groups. This study sheds light on persistent differences in residential mobility and housing patterns among immigrants and their descendants in Europe and contributes to a better understanding of the role of the country context in assimilation of housing behaviour and perpetuating housing inequalities.

## Introduction

A large body of migration literature examines residential mobility and housing of immigrants (Borjas, 2002; Catney & Finney, 2016; Clark & Drever, 2000) or ethnic minorities (Alba & Logan, 1992; Bonvalet et al., 1995). Research shows that residential mobility rates differ between migrant and native populations, with findings of higher and lower mobility depending on the context (Clark & Drever, 2000; Finney, 2011). A common finding is that immigrants are less likely to own their property than natives (Acolin, 2019; Davidov & Weick, 2011; Drever & Clark, 2002; Finney & Harries, 2015; Gobillon & Solignac, 2020; Shankley & Finney, 2020).

For migrants, housing can be an indicator of acculturation to the behaviours and preferences of the majority population and thus integration into mainstream society (Gordon, 1964). Residential mobility and housing tenure (renting vs ownership) reveal patterns of spatial assimilation, upward mobility, or persistent exclusion. Housing tenure, particularly homeownership, is a signal of economic security (Dewilde, 2008) and social belonging (Leviten-Reid & Matthew, 2018) and is widely linked to health and life satisfaction (Angel & Gregory, 2023). Thus, understanding how patterns in housing and residential mobility differ across origin groups, generations, and countries provides important insights into how integration processes unfold under varying institutional contexts.

Previous research suffers from two major shortcomings. First, most studies focus on the first generation (i.e., immigrants) meaning little is known about the experiences of the descendants of immigrants (i.e., second generation). However, investigating whether and how experiences change across generations is critical to understand factors facilitating or hindering migrant assimilation. According to the spatial assimilation theory, as time spent in the host country increases, immigrants’ residential mobility patterns should become similar to those of the native population (Massey & Denton, 1985), and subsequent generations should behave even more similarly (Alba & Logan, 1992; Myers & Lee, 1998). In contrast, a stratification perspective emphasises differential residential and housing paths for different migrant groups (Alba & Logan, 1992, 1993; Andersen, 2016; South et al., 2005). Second, little is known about differences in residential mobility and housing for different immigrant and descendant groups. Some studies have shown that non-European migrants experience less stable housing, necessitating more residential mobility than European migrants (Lerch, 2012). Non-European migrants are also at a particular disadvantage in terms of housing conditions (Pan Ké Shon & Verdugo, 2015; Safi, 2009; Verdugo, 2011). However, further investigation of the heterogeneity in residential outcomes across origin groups is needed to better understand varying experiences of immigrants and their descendants.

Moreover, residential mobility and housing of immigrants and/or their descendants tends to focus on individual-country contexts. To the best of our knowledge, no study has examined cross-national variations in residential mobility and homeownership among immigrants and their descendants. Studying immigrants’ and their descendants’ residential mobility and housing changes will allow us to understand how differences in housing markets and tenure structures (Kemeny, 2006), and different attitudes towards homeownership and renting in European countries (Mulder & Billari, 2010) shape migrants’ residential and housing patterns. We compare the housing and residential mobility patterns of immigrants and their descendants across five countries: the United Kingdom, France, Germany, Switzerland, and Sweden. These five countries are all historically immigrant receiving nations. Thus, by comparing the experiences of individuals from the same or similar origin countries in different destination countries, we can draw conclusions about the role of the host country context. This will enhance our understanding of the role of housing markets and policies in shaping the housing and residential mobility of migrant populations.

## Background

Two theories have been central to understanding the housing and residential mobility of immigrants and their descendants compared to the native population: spatial assimilation and place stratification (or segmented assimilation). The *spatial assimilation* theory argues that the housing situation of immigrants will improve the longer they spend in the destination country (Alba & Logan, 1992; Myers & Lee, 1998) because over time immigrants are likely to have migrated to more advantaged areas (Adelman et al., 2003; Alba et al., 1999, 2000). This theory is derived from the assimilation theory which expects immigrants to display similar patterns to those of the native population as time spent in the host country increases (Alba & Nee, 2003). For subsequent generations the mobility patterns and housing situation are expected to be indistinguishable from that of the native population. *Place stratification or segmented assimilation* theory proposes persistent disparities in residential mobility and residential outcomes between immigrants and natives. In some migrant groups, spatial assimilation may decline rather than increase across successive migrant generations (Portes & Zhou, 1993; Zhou, 1997).

Explanations of why stratification might occur include the limited resources of immigrants which can reduce their ability to relocate and/or become homeowners (Bertocchi et al., 2023; Halliday, 2018), policies which may restrict immigrants’ access to mortgages or social housing, and discrimination which can limit the housing choices of immigrants or ethnic minorities^1^ (Lukes et al., 2019; Massey & Denton, 1985; Özüekren & Van Kempen, 2002). Beyond these structural barriers, minorities may exhibit specific preferences including a desire to live in proximity to ethnic networks which decreases the propensity to move (Boschman & van Ham, 2015). Moreover, their attitudes towards homeownership may be different to that of the majority population (Huber & Schmidt, 2022). These differences in norms can also persist over time and across generations (Portes & Zhou, 1993; Zhou, 1997).

### Residential Mobility and Housing Among Immigrants

Previous studies in various national contexts have shown that residential mobility rates differ between immigrants and natives. Some studies find that immigrants tend to be more mobile than native-born individuals. This was found in Germany (Clark & Drever, 2000), Switzerland, (Lerch, 2012), and Sweden (Rephann & Vencatasawmy, 2000). In the UK, immigrants had similar mobility rates to those of the native population (Mikolai & Kulu, 2024) whereas studies focusing on ethnic minorities have found lower mobility rates among ethnic minorities (Finney, 2011). Residential relocation can be an outcome of poor socioeconomic standing and unstable tenure in the rental market which necessitates repeated relocations (Desmond et al., 2015). However, it can also be a positive outcome related to education and employment transitions or as part of family formation (Bonvalet & Brun, 2002; Smits & Mulder, 2008).

Immigrants also have lower homeownership rates than natives. This was found across North America (Alba & Logan, 1992; Borjas, 2002; Friedman & Rosenbaum, 2004; Haan, 2007), and many European countries including the Netherlands (Bolt & Van Kempen, 2002; Zorlu & Mulder, 2008), Germany (Clark & Drever, 2000; Constant et al., 2009; Davidov & Weick, 2011; Drever & Clark, 2002), France (Acolin, 2019; Fougère et al., 2013; Gobillon & Solignac, 2020; Lévy-Vroelant, 2014; McAvay, 2018; Verdugo, 2011), Sweden (Bråmå & Andersson, 2010; Christophers & O’Sullivan, 2019), and the UK (Darlington-Pollock & Norman, 2017; Finney & Harries, 2015; Mikolai & Kulu, 2024; Shankley & Finney, 2020).

Gaps in homeownership are not consistent across origin groups. For example, in France, immigrants from Sub-Saharan Africa and North Africa display the lowest homeownership rates, whilst European and South-East Asian immigrants are more similar to natives with Turkish-born immigrants taking an intermediate position (McAvay, 2018). Immigrants from Africa are also more likely to live in social housing than natives (Lévy-Vroelant, 2014; Verdugo, 2011).

In Sweden, North African and Sub-Saharan African immigrants have low homeownership rates, whereas Western European immigrants display levels similar to the native Swedes (Bråmå & Andersson, 2010). In Switzerland, homeownership rates vary considerably according to the nationality of household members: native households are the most likely to live in an owner-occupied home, with households headed by foreign-born individuals the least likely (SFSO, 2023). In Germany, no significant differences were found across origin groups. Turks, ex-Yugoslavians, Southern Europeans, and Eastern Europeans had similar transition rates into homeownership as natives (Davidov & Weick, 2011).

In the UK, studies tend to distinguish groups by ethnicity rather than place of birth, generally finding that there are disparities between the White British and minority groups (Shankley & Finney, 2020). South Asians, especially Indians, display high levels of homeownership, whereas households of African and Caribbean origin are more likely to live in social housing. Private renting is most common among the Other White and Arab ethnic minority groups (Finney & Harries, 2015). Immigrants from Europe and India were most likely to move to homeownership or private renting whereas those from Bangladesh, the Caribbean, and African countries were most likely to move to private and social renting (Mikolai & Kulu, 2024).

Differences in mobility rates and housing tenure between migrants and natives can be explained by several factors (Clark & Huang, 2004; Clark & Withers, 2009; Lacroix & Zufferey, 2019). First, immigrants have more limited economic opportunities compared to natives. Upon arrival they may have constrained financial resources and face barriers such as language proficiency and discrimination in the job and housing market (Diaz-Serrano & Raya, 2014). These factors can impact their ability to move (South et al., 2005) leading to lower residential mobility rates. However, this situation may also lead to less stable housing trajectories implying repeated moves and resulting in elevated residential mobility rates, especially shortly after arrival (Andersson, 1996; Clark & Drever, 2000; Fischer & Malmberg, 1997).

Selection effects and the reason for migration may contribute to explaining wealth differences between immigrants and the different residential patterns observed (Chiswick, 1999). According to the adjustment hypothesis, long-distance moves, including international migration, are often followed by short-distance moves due to the imperfect information and initial low resources, meaning that the first dwelling is usually a short-term accommodation (Clark & Withers, 2007). Preferences to be located close to established immigrant communities with access to cultural amenities and social networks may also influence immigrants’ propensity to move (Özüekren & Van Kempen, 2002). Additionally, decisions to not transition to homeownership may be related to different cultural preferences between groups, where some may place less value on assets in the destination country and invest in their home country (Huber & Schmidt, 2022). These preferences can be further fuelled by differences in return intentions (Owusu, 1998). Comparing residential behaviour and outcomes of migrants across different destination contexts, provides the opportunity to uncover more about these mechanisms and how the policy context and selection of immigrants might lead to diverging outcomes both within and between countries.

### Residential Mobility and Housing Among the Descendants of Immigrants

Less evidence is available on the descendants of immigrants and whether their residential mobility and housing patterns differ from those of the natives or from immigrants. Classical assimilation theory would expect to find smaller (or no) differences in residential mobility between immigrants’ descendants and natives (Alba & Logan, 1992; Myers & Lee, 1998). Descendants of immigrants are exposed to the cultural norms of the host country and subsequently do not face the same barriers that their parents have such as lower language proficiency, or limited knowledge about how housing markets operate (Alba & Nee, 1997; South et al., 2005). However, descendants often remain disadvantaged in terms of socioeconomic status, which can limit their access to mortgages and rental properties compared to natives (Meurs et al., 2006; Portes & Zhou, 1993). They may also maintain preferences around living in close proximity to co-ethnics (Özüekren & Van Kempen, 2002).

Moreover, descendants of immigrants may experience limited access to high quality education and services (Crul & Vermeulen, 2003), which may influence their residential mobility patterns. They often have lower employment levels than the native population (Meurs et al., 2006) which can negatively impact their ability to move upwards on the housing ladder and access mortgages. Furthermore, direct discrimination exists in some housing markets where foreign-sounding names seeking a new property are less likely to be invited for a viewing (Auer et al., 2019), this can effect immigrants and their descendants (Quillian et al., 2020) and may lead to persistent differences in residential mobility between the descendants of immigrants and natives.

Few studies compare the residential outcomes of the descendants of immigrants to natives in terms of tenure type, often because the descendants are too young to consider the transition to homeownership. As this generation is becoming more established, research on the housing experience of this group is becoming possible and important. The evidence that is available on the housing experiences of descendants finds that the gap in homeownership rates between the descendants and the native population is smaller compared to the gap between natives and the first generation in France (Acolin, 2019) supporting the assimilation hypothesis. Descendants of immigrants in France, especially those with one native French parent (2.5 generation), are more likely to be homeowners (McAvay, 2018) and less likely to live in social housing (Acolin, 2019) than immigrants. However, other French studies provide evidence of segmented assimilation. Children of non-European immigrants experience higher levels of stratification than other groups, with continued significant differences in housing tenure. This is especially found amongst the descendants of immigrants from Africa and Turkey (Pan Ké Shon, 2011).

UK-born ethnic minority individuals (who can be assumed to be descendants of immigrants) are less likely to experience housing disadvantage than immigrants (Lukes et al., 2019). Although, a large share of UK-born Bangladeshi and Black African individuals experience housing deprivation (Lukes et al., 2019). The propensity of moving to homeownership is larger among the descendants of immigrants than among immigrants, but differences among certain origin groups persist. Individuals of Caribbean, African, and Bangladeshi origin were less likely to move to homeownership and more likely to move to social housing than other groups (Mikolai & Kulu, 2024). Other UK studies tend to use ethnic groups rather than country of birth and migrant generation which makes it impossible to observe intergenerational changes, however Black Caribbean, Indian, Pakistani and Bangladeshi immigrants have a long history in the UK meaning that much of the results of these ethnic disparities are influenced by UK-born minorities. Disparities in terms of tenure exist with lower rates of homeownership for Black African and Black Caribbean groups compared to natives, these groups are also overrepresented in social housing. Other groups, such as Indians and Pakistanis have similar homeownership levels to that of the native population (Shankley & Finney, 2020).

### Cross-National Differences and Contextual Background

#### Differences in Levels of Residential Mobility and Homeownership

Residential mobility varies significantly across European countries, suggesting that the national context can be a factor in differentials between population subgroups. Residential mobility is relatively high in North-western Europe and is much lower in Eastern and Southern Europe (Causa & Pichelmann, 2020). Across the five countries in this study, Sweden and Switzerland have the highest (annual) residential mobility rate (above 35%). The UK and France have a mobility rate between 25 and 35%. Whilst Germany has slightly lower mobility rates (around 22%) (Causa & Pichelmann, 2020).

Homeownership rates also differ cross-nationally. The propensity to live in an owner-occupied dwelling is relatively high in the UK. In contrast, Germany has the lowest rate of owner-occupied housing in Western Europe, with France, Sweden, and Switzerland in-between (Norris & Winston, 2012). Residential mobility and homeownership are inversely related in most countries due to homeowners being the least mobile tenure group (Bonvalet & Brun, 2002; Thomas et al., 2016).

#### Differences in Housing Tenure Structures and Homeownership Regimes

Cross-national differences in residential mobility rates are partly due to differences in institutional arrangements (Borg, 2015; Kemeny, 2006; Mulder & Billari, 2010; Skifter Andersen et al., 2016). Residential mobility levels as well as homeownership rates can vary with the changing composition of the population, the tenure structure of the housing market, and economic conditions in the country (Clark & Drever, 2000).

Tenure structures and housing policies differ across countries (Balchin, 2004; Mikolai et al., 2019). Four homeownership regimes can be distinguished based on the share of owner-occupied housing and access to mortgages (Mulder & Billari, 2010). Most countries in this study (UK, Germany, Switzerland, and Sweden) belong to the ‘career homeownership’ regime where the share of homeowners varies between 30 and 70%. In other words, whilst mortgages are widespread owning is not necessarily the norm and renting is an acceptable alternative. France however is an example of the ‘elite homeownership’ regime, where access to mortgages is more limited (Mikolai et al., 2019; Mulder & Billari, 2010). This means that homeownership needs to be financed from savings, family help, or inheritance.

Two types of renting generally dominate the housing system in Europe, private rental, and social rental. The private renting sector tends to command market-rate rent and is profit driven, whilst the social renting sector is subject to government regulations and is often intended for lower-income or vulnerable households. Two types of rental systems have also been distinguished (Kemeny, 2006). In a dualist rental system, the rental sector consists of an unregulated, generally small private rental sector and a tightly controlled state-regulated rental sector often referred to as social housing (Kemeny, 2006). Social housing is often supported by governments via subsidies. The UK and France have dualist rental systems (Kemeny, 2006). Compared to other advanced economies the UK has a relatively large public rental sector and a relatively small private rental market. Whilst homeownership is the norm, in 2023 private and social renters comprised 20% and 17%, respectively, of households (Office for National Statistics 2023). This is similar in France, where social housing is considered as a crucial element of the housing supply (Lévy-Vroelant, 2014) and represented 18% of French households in 2021 (Ministere de la Transition Ecologique et de la Cohesion des territoires 2022) with around 25% of households being private renters.

In an integrated rental system (Kemeny, 2006), the non-profit rental sector competes on the same terms as the for-profit rental sector and non-profit renting is accessible to the public. This reduces differences in prices between both sectors. Germany, Switzerland, and Sweden have integrated rental systems (Kemeny, 2006). In Germany, market reform has reduced government involvement in the rental sector and cut back housing subsidies to allow market mechanisms to operate (Heeler, 1994; Tomann, 1990). In 2023, about 48% of the population lived in an owner-occupied dwelling, whereas 52% lived in rented accommodation, most of which were private rentals (Eurostat, 2024). In Switzerland, there is a variety of different non-profit providers which represent a small proportion of the stock (20%) and thus do not strongly influence the rental market (Kemeny, 2006). In addition, given that the share of homeowners is especially low, the Swiss population strongly relies on the rental market. In Sweden, municipal housing companies are an important form of housing (Skifter Andersen et al., 2016). This is not strictly social housing but is non-profit. These go alongside for-profit-providers and thus have a leading role on the rental market in terms of rent-setting and availability (Kemeny, 2006). In 2023, 37% of the Swedish population lived in owner-occupied dwellings and 24% lived in tenant-owned apartments, which are effectively owner-occupied dwellings in purpose built apartment buildings (Statistics Sweden, 2024a). The remaining 39% of the population lived in rental properties, of which a little over half were held by private corporations and the remainder by municipal housing companies.

These differences in rental systems and homeownership regimes influence the entire population’s housing choice and mobility. Previous studies argue that integrated rental systems promote lower homeownership rates (Borg, 2015; Voigtländer, 2009). Moreover, access to homeownership is likely to be especially limited in countries where homeownership is either not the norm or access to mortgages is limited. It is likely to be even more limited for migrants compared to the native population. Social housing applications can be complex and for immigrants lower language proficiency can make it more difficult to move to, and between, social housing properties.

Overall, the policy and institutional context that immigrants find themselves in can determine housing access. Access to social housing might be limited based on immigrant status, for example permanent residency may be required, such as in the UK (Lukes et al., 2019). This would have implications for groups with more recent migration history who may be forced into the private rental sector. Similar restrictions apply for the French social housing market (Lévy-Vroelant, 2014, 2015). The extent to which discrimination can affect housing allocation and access for immigrants is also a condition of the policy environment, with greater scope for discrimination the more decentralised policy is (Skifter Andersen, 2012). Countries such as Sweden have more protection to prevent discrimination in these institutions compared to, for example, Germany where housing providers have been observed as replicating socio-inequalities (Hanhörster & Lobato, 2021; Skifter Andersen, 2012).

#### Differences in the Composition of the Immigrant Population

Evidence has shown that immigrants from higher income countries are more likely to have the resources to enter homeownership compared to those from lower income countries (Borjas, 2002). The legal pathway through which migrants have arrived can play a role in housing trajectories (Zorlu & Mulder, 2008). Specific barriers affect migrants arriving under specific schemes, e.g., migrants seeking asylum have more limited access to homeownership than other types of migrants which renders them especially disadvantaged in multiple aspects of social integration, including the housing market (Amuedo-Dorantes & Mundra, 2013; Zorlu & Mulder, 2008).

Therefore, the composition of migrant populations in different countries is a likely factor in the housing and residential mobility differences observed. In Western Europe, post-war migration in the 1950s and 1960s was primarily driven by labour demand. The UK and France received migrants from former colonies—India, Pakistan, and the Caribbean for the UK (Dale & Ahmed, 2011), and North Africa, West Sub-Saharan Africa, and Vietnam for France (Algan et al., 2010). Meanwhile, Germany and Switzerland implemented guest-worker programs to address labour shortages, recruiting migrants from Southern Europe, Yugoslavia, and Turkey (de Haas et al., 2019; Semyonov & Raijman, 2021). By the 1970s, economic downturns led to the end of guest-worker initiatives (Castles, 1986) and shifted migration patterns toward family reunification across Western Europe (Peach, 2006; Semyonov & Raijman, 2021). Additionally, political turmoil in South Asia, the collapse of the Soviet Union and former Yugoslavia, and German reunification spurred asylum-seeker flows into Western Europe. Across Western Europe, intra-EU mobility has also played a significant role in the migrant composition of the population. Since the 2004 EU expansion, migration from Eastern Europe—especially Poland—has been substantial. In the UK, this flow from the EU has declined since Brexit, though European migrants remain a sizable group.

Migrants comprise a significant share of the population in all the study countries, with origins reflecting past labour and colonial ties. France’s immigrant population primarily comes from North Africa (Algeria, Morocco, Tunisia) and Southern Europe (Portugal, Italy, Spain) (INSEE, 2024). Germany’s largest migrant groups originate from Turkey and Eastern Europe (Algan et al., 2010), while Switzerland’s migration flows mainly involve EU and EFTA nationals, particularly from Germany, Italy, and France, alongside third-country migrants from Kosovo and Ukraine (Federal Statistical Office, 2024). In the UK, the most common foreign-born populations come from India, Poland, Pakistan, and the Republic of Ireland (Office for National Statistics 2022).

Sweden’s migration history differs somewhat, with a stronger emphasis on refugee migration which has seen major inflows from Syria, the former Yugoslavia, and Iraq (Statistics Sweden, 2024b). Additionally, labour migration from Yugoslavia, Greece, and Turkey was prominent in the post-war period, the 1970s saw an influx of asylum seekers from Ethiopia, Lebanon, Chile, and Iran (Christophers & O’Sullivan, 2019).

## Hypotheses

Based on the theoretical background and previous studies, we form the following hypotheses.

**H1. Differences Between Immigrants and Natives**

*H1a*. We expect immigrants to have higher rates of residential mobility than natives, especially among groups experiencing more precarious housing conditions.

*H1b*. We expect immigrants to be less likely to move into homeownership and more likely to move into private or social rental tenure compared to natives.

*H1c*. We expect variation in outcomes by country of origin, with non-European immigrants being less likely to access homeownership and more reliant on rental housing compared to European-origin groups.

*H1d*. Consequently, we expect non-European immigrants to be overrepresented in the social rental sector compared to European immigrants.

**H2. Second Generation Immigrants (Descendants)**

*H2a*. In line with assimilation theory, we expect the residential mobility levels of immigrants’ descendants to converge towards those of natives.

*H2b*. Similarly, we expect homeownership transitions to increase for descendants compared to the first generation, especially among those of European origin, approaching levels observed among natives.

*H2c*. However, following the segmented assimilation hypothesis, we anticipate persistent disparities for some non-European descendant groups—such as those of African or Middle Eastern origin—who may show lower transitions into homeownership and higher dependence on social renting compared to other second generation groups.

**H3. Role of National Context and Housing Regimes**

*H3a*. In countries with integrated rental regimes (e.g., Germany, Sweden), we expect lower transition rates into homeownership across all groups compared to countries with dual regimes (e.g., the UK, France).

*H3b*. Accordingly, residential mobility is expected to be higher in countries with more accessible and dynamic rental markets, regardless of origin.

**H4. Structural Constraints and Migration Selectivity**

*H4a*. In countries with limited mortgage access or elite homeownership regimes (e.g., France), non-European immigrant and descendant groups are expected to face stronger barriers to entering homeownership.

*H4b*. Such constraints may lead to higher reliance on rental housing, but not necessarily to higher mobility. As the limited or segmented social housing supply suppresses the opportunity to move.

## Data and Methods

### Data

We use harmonised data from five nationally representative longitudinal datasets: the UK Household Longitudinal Study (UKHLS); the French Permanent Demographic Sample (PDS); the German Socio-Economic Panel (GSOEP); the Swiss population register combined with the Structural Survey; and a 5% random sample of the Swedish population register.^2^ These datasets provide comparable and detailed information on residential mobility and housing tenure over time. We observe individuals between age 15 and 59. Due to differences in the availability of data, there are some differences in the period of observations of residential mobility. We use data from 2010 to 2019 for the UK and Germany. For France, we observe individuals’ residential mobility from 2011 to 2019. For Sweden, we observe individuals from 2010 to 2016, while for Switzerland, we study individuals from 2010 to 2014. These differences in time periods were necessary given the available data, but this should be kept in mind when interpreting the results as possible changes in real estate prices, access to credit, or social housing availability may have influenced residential mobility or tenure transitions differently across countries.

To ensure comparability across data sources, we analyse annual data and define a residential change as a change in residence (identified by a change in the dwelling code, neighbourhood, municipality, region of residence, or a residential move between two years).^3^ For the UK and Germany retrospective information is available from when individuals enter the panel and are asked each year whether they have moved since the last interview and if so to report the year and month of their move. For France, individuals’ residential mobility can be inferred if there is a change in the dwelling of residence from one year to another. In other words, residential mobility is based on the information provided by all individuals on a yearly basis in their fiscal records, with information on housing tenure also available. Annual data on housing changes is available in both the Swiss and Swedish register data, however, information on housing tenure is not collected in Switzerland.

### Methods

To compare the patterns of residential mobility and housing tenure changes across countries and population subgroups, we use the count data approach (Kulu et al., 2021). This entails preparation of an occurrence-exposure (or event-time) table, defined by cross-classifying over a set of time intervals and variable categories (Hoem, 1975; Preston, 2014). The cells of the resulting table include the number of events (e.g., residential changes) denoted as ${E}_{jk}$ and risk time (i.e., person-years) denoted as ${R}_{jk}$ for each possible combination of covariate categories for each age group $j$ and variable category $k$. For each cell, the hazard or rate denoted as ${\mu }_{jk}$ is obtained as the ratio of the number of events to the risk time:

1 $${\mu }_{jk}= {E}_{jk}/{R}_{jk}$$

We treat ${E}_{jk}$ as the realisation of a Poisson random variable. The expected number of residential changes is the product of the hazard of residential change and exposure time. We estimate a series of Poisson regression models on the pooled occurrence-exposure dataset for five countries. The models can be expressed as follows:

2 $$\text{ln}{\mu }_{jk}= {\alpha }_{j}+ {X}_{k}{\prime}\beta$$

where ${\alpha }_{j}=\text{ln}{\mu }_{jk}$ measures the hazard of residential changes by age (the ‘baseline’), ${X}_{k}{\prime}$ is a vector of the covariates, and $\beta$ represents a vector of the parameters to measure their effects. This approach is equivalent to estimating piecewise constant event history models with categorical variables and to estimating discrete-time logit models on discrete-time data (Holford, 1980; Laird & Olivier, 1981).

We estimate two models. First, we estimate the risk of a residential change by origin group among immigrants, immigrants’ descendants, and native individuals in all countries (Model 1). Second, we estimate the risk of a move to different housing tenure types: (i) homeownership, (ii) social renting, or (iii) private renting (Model 2). This is done by applying a competing risk framework where the occurrence-exposure table is disaggregated further such that the event of interest is moving to each specific tenure type by age (the ‘baseline’). Since Switzerland does not have information on housing tenure, it is excluded from this model. Moreover, we must exclude those with missing information on destination tenure, which is a small proportion in each country. We present results for men and women together; results are similar when stratified, due to the propensity for moves to be undertaken by entire households.

### Variables and Modelling Strategy

The main independent variable is the origin group, incorporating the native population, first (G1) and second generation (G2) groups in all countries. Immigrants are defined as persons born outside of the country (and without French citizenship in France).^4^ The descendants of immigrants are those who were born in the host country to at least one immigrant parent or those who were born in a foreign country but migrated to the host country as children, i.e., before the age of 16.^5^ Finally, the natives are individuals who were born in the country (and with French citizenship in France) whose parents were also born in the country (and with French citizenship in France). We further distinguish immigrants and their descendants by origin. A detailed list of the countries included in each origin group is in Appendix A, Table 1.

We further control for sex and age groups (15–19, 20–24, 25–29, 30–34, 35–39, 40–49, and 50–59). Additionally, we include a control for parity (childless, 1 child, and 2 + children) and partnership status (single, partnered, and separated/widowed). Partnered individuals include those who are married, cohabiting, or in a civil partnership. Employment status is categorised as employed, unemployed, inactive, or unknown. This variable is either self-reported (in the UK and Germany) or based on the information provided on earnings (in France, Switzerland, and Sweden). Lastly, we control for level of education (low, medium, or high). In France there is a high proportion of missing values for education, therefore we proxy education using the tercile of household standard of living, i.e., income by unit of consumption which is highly correlated with education (when known) in France.^6^ Model 2 includes all previous controls and additionally includes the original tenure state that an individual is in (owner-occupied, social renting or private rental). Descriptive statistics for the number of residential moves and person years can be found in Appendix B, Table 2. Moreover, the number of residential moves to different housing tenure types by origin group can be found in Appendix B, Table 3.

## Results

### Residential Mobility Patterns

**Fig. 1:**
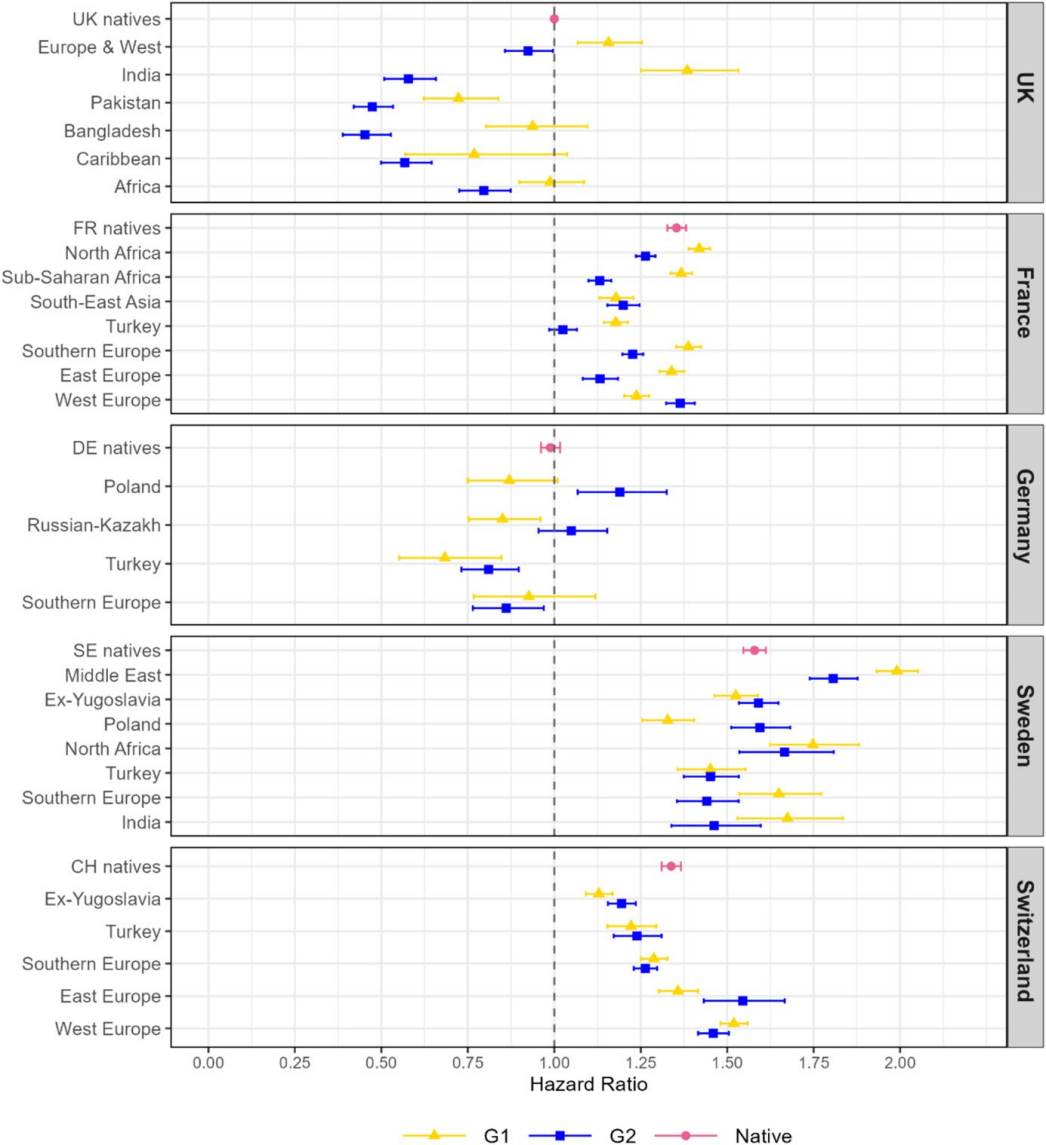
Hazard ratios of a residential move by origin group and generation in the UK, France, Germany, Switzerland, and Sweden. *Source* Authors’ own calculations using data from the UK household longitudinal study (UKHLS), the French permanent demographic sample (PDS), the German socio-economic panel (GSOEP), the Swiss population register, and a 5% random sample of the Swedish population register. The data analysed is for the period 2010–2019 for the UK and Germany; 2011–2019 for France; 2010–2014 for Switzerland, and 2010–2016 for Sweden. Notes: The model is adjusted for sex, age, education, partnership status, parity, and employment status. Whiskers indicate 95% confidence intervals compared to the baseline hazard of natives in the UK. Full regression results are reported in Appendix B, Table 4

Figure 1 shows the hazard ratios (HR) of a residential change for immigrant and descendant groups compared to UK natives. First, we can observe differences between countries. The UK and Germany have similar rates of residential change whereas Switzerland, France, and Sweden have higher risks of moving. This is in line with what we know from the literature about cross-national differences in residential mobility (Causa & Pichelmann, 2020). Heterogeneity between groups appears to be most prominent in the UK and Sweden compared to the other countries, yet all show variation across origin groups.

In all countries bar Germany, there are G1 groups with higher likelihoods of moving compared to natives and others with lower. In the UK immigrants from European and Western countries and India have significantly higher risks of moving than natives (HRs: 1.25 and 1.53 respectively). Whereas immigrants from Pakistan is significantly lower (HR: 0.84). In France, most immigrant groups have a similar risk of experiencing a move to the French natives (HR: 1.38), except those born in Turkey and South-East Asia where the risks are significantly lower (HRs: 1.21 and 1.22 respectively). In Sweden, immigrants born in the Middle East have the highest risk of moving of any group in the study (HR: 2.05). In Switzerland, Western European migrants have the highest risk of moving compared to natives and those from former Yugoslavia have lower risks.

There is no consistent pattern of assimilation between generations. The UK consistently shows lower mobility for G2 compared to both natives and G1, the exception being the G2 of Europe and Western background. France is also similar in this regard; only Western European descendants have higher residential mobility than the G1 group. By contrast in Germany the opposite is the case with G1 groups having lower mobility than G2. Polish descendants have mobility even above that of native Germans (HR: 1.33). For Sweden and Switzerland, the differences between the G1 and G2 were smaller. In Sweden, Polish G2 groups (HR: 1.68) had a higher risk of moving than the Polish G1 (HR: 1.40) and the same trend was found for Eastern European (many of whom from Poland) in Switzerland.

Both the UK and Sweden have migrants from India, who display similar mobility rates in the two countries. Among the second generation, the descendants of Indian immigrants residing in Sweden seem to be more mobile compared to those in the UK. France and Sweden have a significant share of immigrants and descendants from North Africa who are more mobile in Sweden than in France. However, the colonial ties of North Africans in France and Indians in the UK may mean these subgroups are less comparable to those of the same origin elsewhere in Europe, as the result is a highly different age structure and different characteristics on arrival due to selection (Chiswick, 1999). Individuals of Turkish origin are similar across all contexts as having lower likelihood of moving compared the native population, and most other immigrant groups. Generally, this persists between generations and in the case of France becomes even lower for the G2. Western European immigrants are prevalent across the contexts too, although not always identical with respect to the specific origin countries, the results indicate that mobility for the G1 in Switzerland is slightly higher and in France slightly lower, but amongst descendants there is convergence to native levels.

### Destination Tenure

**Fig. 2:**
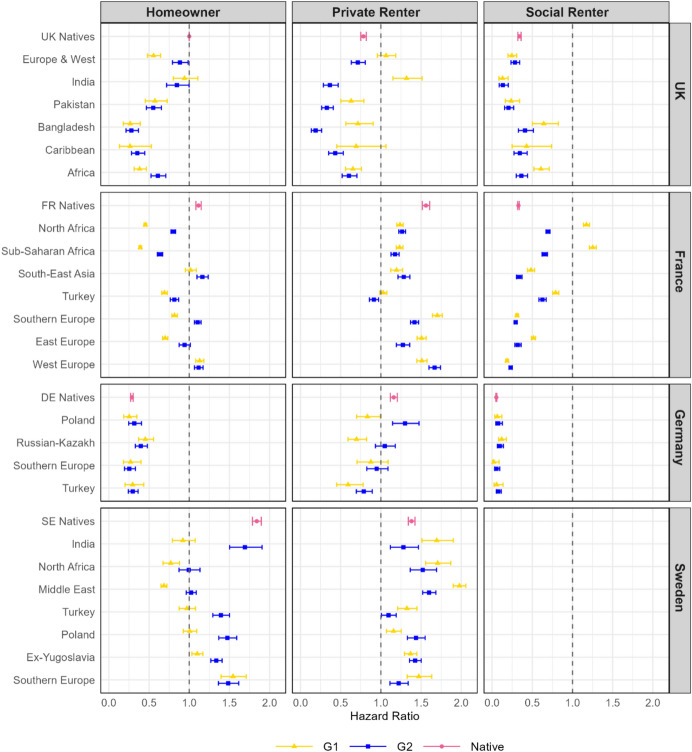
Hazard ratios of a residential move to different tenure types by origin group and generation in the UK, France, Germany, and Sweden. *Source* Authors’ own calculations using data from the UK household longitudinal study (UKHLS), the French permanent demographic sample (PDS), the German socio-economic panel (GSOEP), and a 5% random sample of the Swedish population register. The data analysed is for the period 2010–2019 for the UK and Germany; 2011–2019 for France; and 2010–2016 for Sweden. *Notes:* The model is adjusted for age, sex, education, partnership status, parity, employment status, tenure at origin and time period. Whiskers indicate 95% confidence interval. The baseline hazard is a native moving to a homeownership in the UK. For Sweden we display all types of rentals as “private renter.”

Next, we study differences in the type of housing tenure that individuals move to by origin group and migrant generation. Figure 2 shows the results; the baseline hazard represents the risk of moving to homeownership for UK natives. It is important to note that these estimates do not necessarily mean that the tenure has changed since lateral moves between the same tenure state are commonplace. Switzerland is excluded from the analysis as there is no information on tenure. Since social renting is not identifiable in Sweden all renting is considered private renting.

When comparing the risks of a move among natives across the four countries, natives in Germany have a low risk (HR: 0.30) while natives in France and Sweden have a higher risk of moving into a homeownership (HR: 1.14 and 1.85 respectively) compared to the risks of UK natives. This is in line with previous studies showing different homeownership levels across these countries (Andrews & Sánchez, 2011).

First generation migrant groups tend to have less mobility towards homeownership than natives but there is variation between groups. The biggest gaps between natives and immigrants when it comes to homeownership access is in Sweden and France, particularly for North and Sub-Saharan Africans in the latter. There are a few instances where homeownership moves are at the same rate as the native population, Indian born immigrants in the UK and South-East Asian and Western European migrants in France. This is also the case in Germany which has little variation in homeownership risks and social renting risks, a factor of the specific market that exists there.

The importance of the rental sectors for the housing needs of minority groups becomes clear with these gaps to homeownership evident. In France, North African and African immigrants also show elevated risks of social renting (HR: 1.21 and 1.30 respectively), reinforced by the limited homeownership transitions observed. There are also divergences between immigrant groups, Indian immigrants in the UK have a higher risk of moving to private rental compared to natives. This contrasts with Bangladeshi and African immigrants who have a much higher likelihood of moving to social renting than natives (HR 0.82 and 0.71 vs HR 0.36 for natives). Variation in Sweden is also found with G1 immigrants from India, North Africa, and the Middle East more likely to move to private renting compared to the native Swedes, whilst Polish immigrants have a significantly lower risk of that type of transition.

Our results also indicate that the descendants of immigrants typically show more transitions to homeownership than the G1. This could partly be explained by age structure differences and life course transitions including entry to homeownership happening prior to the observation window for the immigrant generation. However, it could also indicate a level of acculturation to native customs and consumer behaviour. Despite this there are few examples of convergence to natives, amongst G2 groups it is typically those of European and Western backgrounds who move to homeownership at similar rates to natives in all country contexts. G2 groups of non-Western origins which show similar rates of transition to homeownership as natives would be Indian G2s in Sweden and the UK and South-East Asian G2s in France. For other non-Western groups, despite evidence of some convergence towards native levels there are still observable differences with an increased likelihood of a move to social housing, particularly for the groups where G1 are also highly represented in social housing. For example, North African, Sub-Saharan Africans and Turkish in France and Bangladeshis and Africans in the UK.

Comparing similar origin groups in different contexts we see that immigrants from North Africa in France have a relatively high risk of moving to social renting, they are less likely to move to homeownership compared to their counterparts living in Sweden, but in both destinations and across generations they experience a disadvantage in terms of homeownership access compared to natives. Compared to natives Turkish immigrants and their descendants are found to have similarly low, or lower, risks of moving to a private rental. However, this is not reflected in particularly high rates of moving to homeownership. Notably for the Turkish descendants those in Sweden have a much higher risk of moving to homeownership compared to Turkish descendants in other countries, but it is still lower than the risk of natives.

## Discussion

Across five countries, the UK, France, Germany, Switzerland, and Sweden, this study analysed differences in residential mobility and tenure across migrant generations, origin groups, and country contexts. We first studied the risk of experiencing a residential move; we then analysed the risk of moving to different housing tenure types (homeownership, private renting, social renting). Our key contribution is the use of a cross-national study to shed light on the importance of the destination context for the housing assimilation of immigrants and their descendants. To our knowledge this is the first quantitative study which has taken a cross-national approach to study generational changes in housing for those with an immigrant background.

We expected that immigrants would have higher mobility rates than the natives (H1a). We found partial support for this hypothesis, immigrants from India and Europe and Western countries in the UK, those from the Middle East in Sweden, and Western Europeans in Switzerland do have higher mobility than natives. However, in France and Germany immigrants did not show elevated mobility compared to natives and quite often it was significantly lower. These results also show some support for the hypothesis that certain migrant groups will display higher mobility rates (H1c). However, our expectation that elevated mobility for non-European groups was not particularly matched. The examples of elevated mobility amongst non-European immigrants were Indian immigrants in the UK, and Middle Eastern immigrants in Sweden (many of these are Iranian refugees). These groups have specific selection pathways and have a long-standing history in the destination (Chiswick, 1999). Due to this they have accumulated capital to move and enter homeownership. Upon arrival they may have faced barriers to housing and mobility, possibly resulting in ethnic segregation, but over time they have spatially assimilated and left these areas for preferable housing situations. Higher mobility rates for immigrants from Europe and Western countries in the UK and those from Western Europe in Switzerland can also be explained by the relative success of these groups. They face lower barriers compared to other migrant groups, less discrimination, and are more likely to have increased knowledge of the housing market (which is more likely to be similar to the one in their origin country), all of which contribute to gaining more resources to move.

Our expectation of higher mobility risks for immigrant groups, particularly non-Europeans, was not found in France or Germany and mostly not found in Switzerland. This might indicate that, in this period at least, precarity in the housing market is not driving noticeably higher residential mobility for immigrant groups (Clark & Drever, 2000; Lerch, 2012). However, there are several cases where immigrant groups have a lower risk of moving than the majority population, for example Pakistanis in the UK, and South-East Asian immigrants in France. What this could indicate is that the housing markets in different countries are adversely affecting immigrants, possibly due to a mismatch in where the housing stock is located and where immigrants are located, which is preventing mobility from reaching the same level as the natives who are geographically distributed more evenly. Alternatively, it could show that barriers such as discrimination are prevalent and are inhibiting mobility (Acolin et al., 2016; Diaz-Serrano & Raya, 2014; Lukes et al., 2019).

We expected immigrants to be less likely to move to homeownership and more likely to move to social or private renting compared to the natives (H1b). Our expectations were largely confirmed. Almost all migrant groups have a lower likelihood of moving to homeownership than the natives across the study countries. The groups least likely to move to homeownership relative to natives were North African and Sub-Saharan African immigrants in France, those from the Middle East and North Africa in Sweden, and African and Caribbean migrants in the UK. At the same time, these groups are the most likely to move to social (in France and the UK) or private renting (in Sweden), confirming both H1b and H1d that non-Western immigrant groups are the least likely to access homeownership and most likely to move into social housing. We find that some immigrant groups fare better than others and align closely with natives when it comes to moving to homeownership. For example, Indian immigrants in the UK, and South-East Asian and Southern European immigrants in France are more likely to move to homeownership compared to other migrant groups. Again a reflection of better socioeconomic outcomes and wealth accumulation, as well as lower cultural barriers for European migrants (Khoudja & Platt, 2018; Meurs et al., 2006).

In line with the assimilation hypothesis, we expected the descendants of immigrants to have similar residential mobility (H2a) and homeownership rates (H2b) to natives rather than immigrants (Alba & Logan, 1992; Myers & Lee, 1998). However, we also expected to find some persistent differences between some descendant groups (those from non-European backgrounds) and natives (H2c). We expected these groups to have higher residential mobility, lower propensity of moving to homeownership, and a higher risk of moving to a social rental property than the natives. Our results show a decline, or no change, in total mobility rates for most second generation groups compared to the immigrant generation. In the UK and France, this shift also means lower mobility than natives, with the only exception being Western European descendants in France. In Germany, Sweden, and Switzerland, residential mobility of the second generation is like that of immigrants casting doubt on assimilation through generations. Therefore, there is limited support for H2a. However, there are some indications of assimilation regarding the destination tenure, and support for H2b. In France, the descendants of immigrants from North Africa and Sub-Saharan Africa have a lower risk of moving to social renting than their parents’ generation, and the same is true of Bangladeshi and African descendants in the UK. Also, in Sweden, nearly all descendant groups have a higher risk of moving to homeownership than immigrants. However, evidence for the stratification or segmented assimilation perspective remains, as despite the decline in differences in moving to homeownership between natives and the descendants compared to the immigrant generation, we still observe low levels of moving to homeownership and elevated levels of social renting among many descendant groups. This persistent difference is particularly found for non-European descendant groups in the UK, France, and Sweden, confirming H2c.

We expected experiences of residential mobility and tenure to vary based on the housing systems in the destination countries. We hypothesised that integrated rental systems (Germany and Sweden) would display lower rates of moves to homeownership than countries with a dual rental system (the UK and France) (H3a). We find support for this in Germany which has far lower homeownership moves compared to France and the UK. We speculate this down to the overall low levels of homeownership in the country (Norris & Winston, 2012). Renting is viewed as a permanent outcome and thus the incentive to move is lower as the tenure is more secure than in other countries (Kaas et al., 2021), this societal norm seems to be a factor for immigrants and descendants as well as natives. However, rates of moving into homeownership were consistently higher in Sweden across all subgroups compared to other countries. We consequently predicted that these integrated rental sectors would see higher levels of residential mobility compared to those without (H3b), this we did not see support for, Germany as a consequence of strong tenancy rights and long-term renting being the norm displayed the lowest level of residential mobility contrary to the theory.

Our final hypothesis was that some groups among non-European immigrants and their descendants would display especially low rates of homeownership in countries where access to mortgages is limited (H4a). Focusing on France, where there is ‘elite homeownership’ we found that both the first and second generation of North and Sub-Saharan African origin do not move to homeownership to the same extent as other groups, or natives. However, this difficulty did not contribute to overall higher residential mobility due to precarity in the rental market, therefore countering our final hypothesis (H4b). It seems instead that in France the use of social housing by these more marginalised immigrant groups creates an element of stability and is a long term housing solution (Fougère et al., 2013). However, that does not necessarily mean that these homes are adequate for the needs of these groups, and mobility may be lower than it would be because options of social housing are limited.

Our results confirm much previous work from when these contexts have been studied in isolation (Bråmå & Andersson, 2010; Lévy-Vroelant, 2014; Shankley & Finney, 2020; Verdugo, 2011). Our work extends upon these studies by highlighting clearly that differences in the extent of intergenerational assimilation of both mobility and housing tenure exists across destinations. Some countries such as Germany have very few differences between immigrant generations and between the natives, which as we mention is likely driven by the societal norms of a low ownership society, and the integrated rental sector. However, the elite homeownership regime in France sustains high reliance on social renting and low movement into homeownership particularly amongst immigrant groups of North and Sub-Saharan African origin. Importantly, these differences are sustained for the descendants. There is evidence of intergenerational change but there are similarities across Sweden, France, and the UK, in that moves to homeownership are less prevalent for the second generation compared to natives. There are some exceptions, but despite heterogeneity in the rental and housing markets and different migration selection this gap exists throughout Europe.

Disadvantage in access to homeownership permeates through generations, alluding to theories of segmented assimilation and stratification of immigrants and their descendants (Portes & Zhou, 1993). The mechanisms behind this can include the barriers to information regarding the housing market and structural constraints such as the lack of banking and employment history in the destination (Clark & Withers, 2007). Moreover, experiences of residential segregation linked to discrimination have been detailed, for certain groups, in the countries in this study (Andersson et al., 2010; Drever & Clark, 2002; Finney, 2011; Magnusson & Özüekren, 2002; Safi, 2009; Sürig & Wilmes, 2015).

This study contributes to a better understanding of the role of the host country context in perpetuating inequalities in the residential mobility of immigrants and their descendants, which is of importance to policymakers in an increasingly heterogenous Europe. We highlight persistent differences in residential mobility and housing patterns among immigrants, their descendants, and natives in Europe. Policymakers should attempt to alleviate these inequalities by lowering structural barriers, increasing access to credit, and potentially regulating the housing market more. The case of Germany shows far smaller within country differences in both mobility and tenure outcomes. The heavily regulated rental market has made long-term renting a viable alternative to homeownership which can be associated with these lower inequalities (Voigtländer, 2009). This is contrary to France where the elite homeownership regime perpetuates inequalities in housing tenure with high usage of social renting for immigrants and their descendants from North and Sub-Saharan Africa. These groups may see homeownership as unattainable under the current system and continue to pursue social housing as an alternative (Fougère et al., 2013; Lévy-Vroelant, 2014).

Our results do show heterogeneity between different origin groups, suggesting that the destination context alone is not the only mechanism. We found that differences between migrants from other European origins are lower, this group are more likely to have native partners (Hannemann et al., 2018), who may lead navigation of the housing market. However, even without a native partner European migrants face lower barriers to employment and housing opportunities than immigrants from non-Western origins. Some differences between groups could also be explained by differences in duration of residence in the destination country. This may have led to the accumulation of wealth and adaptation to native behaviours over time. Additionally, soon after migration, immigrants’ levels of residential mobility (perhaps more so among some groups than others) may be higher than those of the native population. However, as information on the duration of residence in the host country was not available (at least completely) in France and Switzerland, we were unable to explore the role of this variable, and we leave this issue for future research.

It is important to reflect on the normative assumption this research makes around homeownership as the preferred tenure state. Whilst housing tenure and mobility can be seen as a marker of success and integration (Alba & Nee, 2003; Borjas, 2002; Gordon, 1964), housing is not solely a structural outcome; preferences related to culture are also likely to play a role in housing transitions and residential mobility. The deep social and cultural significance of housing and place may vary between natives, first- and second-generation immigrants, and across different origin groups (Huber & Schmidt, 2022). There could be different expectations due to transnational links to family in the origin which results in less investment in becoming a homeowner in the destination country (Özüekren & Van Kempen, 2002). Mobility may be less desirable compared to goals such as maintaining proximity to family, community networks, or a preference for specific housing arrangements, for example multigenerational households. Thus, it is important to recognise that immobility and remaining in a segregated area can be an outcome of stratification but on the other hand can be a choice to remain embedded in social networks (Alba & Logan, 1992; Portes & Zhou, 1993).

Due to the need for comparable data across countries to allow for creation of the occurrence-exposure table, there is a limit to the covariates that can be included in this study. First, in order to include Switzerland in the analysis of total residential mobility we were unable to control for the current housing tenure, despite an individual’s current housing tenure being a determinant of subsequent mobility (Bonvalet & Brun, 2002). In Appendix B Table 4 is a sensitivity analysis named Model 1b which includes a control for the current housing tenure. The results are impacted by the inclusion of the current tenure, as expected it does lower residential mobility gaps for groups who are not typically homeowners; however, the overall narrative remains the same. Second, it is known that the desire to relocate decreases over the length of time spent in a dwelling as attachment to the property increases (Fischer and Malmberg 2001; Thomas et al., 2016). Ideally, a control for the length of time that an individual has lived in a property would account for this, as it would be particularly relevant for immigrants with more recent histories. However, most datasets do not include this information.

We also advise some caution with interpretation of our results due to compromises made to allow for the comparative research design to be implemented. For example, homeownership is measured at the household level since most datasets do not identify who in the household is the owner. This can lead to an overestimation of the risks of individuals to move to homeownership if they move as part of a household where someone else (e.g., a parent) is the owner. Combining administrative data with other data sources such as survey or qualitative data may help in overcoming this limitation in future studies. Moreover, our results do not show tenure trajectories, we are limited to observing only the type of tenure that an individual moves to, it would be more informative to understand if moves are lateral or upwards on the housing ladder. Whilst the original tenure is controlled for, we recommend that future comparative studies investigate housing tenure trajectories of immigrants and their descendants to gain a better understanding of these dynamics. Lastly, immigrants are known to suffer more overcrowding and worse housing quality than the native population (Gobillon & Solignac, 2020; Lukes et al., 2019), and our normative assumption that it is preferential to move to homeownership rather than a social rental property could be debated when considering measures of housing quality. Future studies should consider distinguishing more housing quality and the suitability of the property.

Residential mobility and tenure have implications for social cohesion and economic security; and act as a signal for how similar migrant behaviours have become to the majority population. This research has found substantial heterogeneity in the residential mobility and housing tenure of immigrants and their descendants across Europe. We have extended previous research by demonstrating the importance of the destination country context and how the housing assimilation of immigrants can vary depending on the environment of the host country. We find evidence of assimilation in homeownership transitions between generations, however convergence to native levels for the second generation is generally found amongst the descendants of Western and European migrants. Those from non-Western origins and their descendants remain at a disadvantage. These findings suggest that segmented assimilation exists for these non-Western groups particularly in France and the United Kingdom. These findings may not only be down to structural factors, and we encourage further research to explore housing intentions and desires of immigrants and their descendants. These future studies should also consider a cross-national perspective to identify how institutional differences and policy contexts can impact immigrant integration, particularly in relation to residential mobility and tenure.

## Appendix A

Data Sources.

For the UK, we use data from Waves 1 to 9 (2009–2019) of the UK Household Longitudinal Study (UKHLS); a nationally representative household panel survey that interviews approximately 30,000 households (~ 51,000 individuals) annually. The UKHLS also includes two immigrant and ethnic minority boost samples (in waves 1 and 6) enabling us to study a large sample of individuals from various origins.

For France, we rely on the Permanent Demographic Sample (PDS) – or Echantillon Démographique Permanent – which was developed by France’s Institut National de la Statistique et des Etudes Economiques (INSEE). It comprises information taken from the official publications of the registry office for births, marriages, and deaths since 1968, along with exhaustive census information from 1968, 1975, 1982, 1990 and 1999. In addition, it contains information from annual fiscal reports from 2011 to 2019 as well as more specific employment information for a subset of employees.

Using the French PDS, the immigrant status of individuals can be determined using information on the country of birth and citizenship at birth. Therefore, we define immigrants as persons born outside of France without French citizenship at birth. Regarding immigrants’ descendants, it is not possible to directly identify their origin in the EDP data. However, because of the availability of parental variables among EDP individuals who were observed as children, a national origin can be assigned to children of immigrants by taking parental country of birth as a proxy for the origin of EDP children. Immigrants’ descendants may have been born in France or migrated as children. Finally, French natives are individuals born with French citizenship whose parents were also born with French citizenship.

For Germany, we use data from the German Socio-Economic Panel (SOEP, version 37); a household panel study of over 19,000 households that started in 1984 and is still ongoing. We use data from all waves (1984–2020) for individuals who reside in West Germany. The data also includes a foreign subsample which enables us to study the patterns of first- and second-generation immigrants from various origin countries.

For Switzerland, we use the population register combined with the Structural Survey – a nationally representative survey conducted every year on at least 200,000 inhabitants. Individuals of foreign origin can be identified with their country of birth and year of arrival. Information on the parents’ origin only indicates whether they were born in Switzerland or abroad without specifying the country of birth. However, the descendants of immigrants can be identified with the country of birth (Switzerland), current nationalities, and the date of acquisition of the Swiss nationality. If the individual has two foreign-national parents, he/she will not have the Swiss nationality at birth.

Lastly, for Sweden, we use a 5% random sample of the Swedish population register (Statistics-Sweden, 2023). This dataset includes information on all individuals with legal residence in Sweden starting from 1968, when digitisation of register records took place. The Swedish register microdata is updated continuously, and it includes information on individuals’ main socio-demographic characteristics (e.g., sex and country of birth), and records of events such as births, civil status, deaths, international migrations (i.e., immigration, and emigration), and internal migrations (i.e., residential mobility). It also includes information on a yearly basis on education, employment, income, and benefits received for all individuals aged 16 and older and information on residential property identification numbers. Additionally, the Swedish microdata includes a “Longitudinal Database for Integration Studies (STATIV),” which provides valuable information including year of arrival in Sweden (for immigrants), municipality of residence, housing tenure, and housing type.

See Table 1.

**Table 1:** List of countries of birth by origin group and country.

Country	Origin group	Countries of birth
UK	Europe and West	France, Germany, Italy, Ireland, Spain, Poland, Cyprus, Turkey, Portugal, Albania, Armenia, Austria, Belarus, Belgium, Bosnia and Herzegovina, Bulgaria, Channel Islands, Australia, Czech Republic, Denmark, Finland, Georgia, Gibraltar, Greece, Hungary, Jersey, Kosovo, Latvia, Lithuania, Malta, Moldova, Norway, Portugal, Romania, Russia, Serbia, Slovakia, Slovenia, Sweden, Switzerland, the Netherlands, Ukraine, Yugoslavia, New Zealand, Canada, USA
	India	India
	Pakistan	Pakistan
	Bangladesh	Bangladesh
	Caribbean	Jamaica, Anguilla, Antigua, Bahamas, Barbados, Cuba, Dominica, Dominican Republic, Guadeloupe, Grenada, Guyana, Haiti, Montserrat, Nevis, St Lucia, St Vincent and the Grenadines, Trinidad and Tobago
	Africa	Kenya, Ghana, Nigeria, Uganda, Algeria, Angola, Benin, Botswana, Burkina Faso, Burundi, Cameroon, Cape Verde, Zaire, Democratic Republic of Congo, Djibouti, Egypt, Eritrea, Ethiopia, Gabon, Gambia, Guinea, Guinea-Bissau, Ivory Coast, Liberia, Libya, Madagascar, Malawi, Mauritius, Morocco, Mozambique, Namibia, Rwanda, Senegal, Seychelles, Sierra Leone, Somalia, Sudan, Swaziland, Tanzania, Togo, Tunisia, Zambia, Zimbabwe
France	North Africa	Algeria, Morocco, Tunisia
	Sub-Saharan Africa	Angola, Botswana, Burkina Faso, Burundi, Cameroon, Central African Republic, Chad, Comoros, Congo, Republic Democratic of Congo, Ivory Coast, Djibouti, Gabon, Gambia, Ghana, Guinea, Guinea Bissau, Kenya, Liberia, Lesotho, Madagascar, Malawi, Mali, Mauritania, Niger, Mozambique, Namibia, Niger, Nigeria, Rwanda, Senegal, Sierra Leone, Somalia, South Africa, Soudan, Eswatini, Tanzania, Togo, Uganda, Zambia, Zimbabwe, Benin, Mauritius
	South-East Asia	Vietnam, Cambodia, Laos
	Turkey	Turkey
	Southern Europe	Greece, Portugal, Spain, Italy, Cyprus
	East Europe	Ukraine, Slovakia, Slovenia, Serbia, Russia, Romania, Poland, Moldova, Macedonia, Lithuania, Latvia, Hungary, Georgia, Estonia, Czech Republic, Croatia, Albania, Armenia, Bosnia and Herzegovina, Bulgaria, Belarus
	West Europe	The UK, Switzerland, Sweden, Norway, the Netherlands, Monaco, Malta, Liechtenstein, Ireland, Iceland, Germany, Finland, Denmark, Austria, Belgium, Montenegro
Germany	Polish	Poland
	Russian-Kazakh	Russia, Kazakhstan
	Southern Europe	Portugal, Italy, Spain, Greece
	Turkey	Turkey
Switzerland	Ex-Yugoslavia	Kosovo, Serbia, Serbia and Montenegro, Croatia, Slovenia, Bosnia and Herzegovina, Montenegro, Macedonia
	Turkey	Turkey
	Southern Europe	Greece, Portugal, Spain, Italy, Cyprus
	East Europe	Albania, Bulgaria, Poland, Romania, Estonia, Latvia, Lithuania, Moldovia, Russia, Ukraine, Belarus, Hungary, Slovakia, Czech Republic
	West Europe	Germany, France, Austria, Belgium, the Netherlands, the UK, Ireland, Iceland, Denmark, Finland, Norway, Sweden, Liechtenstein, Luxembourg, Malta, Andorra, Monaco
Sweden	India	India
	North Africa	Egypt, Algeria, Morocco, Libya, Tunisia
	Middle East	Palestine, Yemen, Lebanon, Syria, Iraq, UAE, Bahrain, Yemen, Jordan, Kuwait, Oman, Qatar, Saudi Arabia, Gaza, West Bank
	Turkey	Turkey
	Poland	Poland
	Ex-Yugoslavia	Yugoslavia, Croatia, Macedonia, Montenegro, Serbia, Slovenia, Bosnia and Herzegovina
	Southern Europe	Andorra, Gibraltar, Portugal, Spain, Greece, Cyprus, Italy, Malta, San Marino, Vatican

## Appendix B

See Tables 2, 3 and 4

**Table 2:** Number of residential moves and person-years by categories of variables. *Source* The UK household longitudinal study (UKHLS), the French permanent demographic sample (PDS), the German socio-economic panel (GSOEP), the Swiss population register, and a 5% random sample of the Swedish population register, authors’ own calculations. The data analysed is for the period 2010–2019

	UK		France		Germany		Switzerland		Sweden	
	Moves	Person-years	Moves	Person-years	Moves	Person-years	Moves	Person-years	Moves	Person-years
*Sex*										
Men	6038	81,333	662,057	6,173,438	5067	67,487	71,406	696,017	92,833	709,766
Women	7763	105,717	685,368	6,183,888	6739	81,211	74,975	718,735	92,143	670,083
*Age*										
15–19	1277	14,595	13,532	310,506	891	18,240	7385	100,354	17,126	154,377
20–24	2750	17,067	182,120	1,128,326	2121	15,531	26,700	123,588	49,884	176,015
25–29	2300	16,251	307,157	1,386,852	2353	14,374	30,236	132,349	37,154	157,369
30–34	2077	19,905	250,654	1,511,235	2182	18,181	24,692	155,308	23,448	146,439
35–39	1664	22,587	181,594	1,545,633	1608	21,182	17,620	167,318	15,874	154,063
40–49	2376	50,731	253,529	3,298,795	2185	47,075	24,911	393,615	25,103	326,204
50–59	1357	45,914	158,839	3,175,979	466	14,115	14,837	342,220	16,387	265,382
*Time period*										
2010–2014	9255	118,633	493,896	5,361,614	6034	77,190	146,381	1,414,752	121,027	985,977
2015–2019	4546	68,417	853,529	6,995,712	5772	71,508			63,949	393,872
*Partnership status*										
Single	7759	73,305	728,359	5,264,680	4862	53,196	84,808	552,705	133,601	807,533
Partnered	4520	92,417	498,035	6,108,309	5723	83,963	49,919	739,182	36,202	464,438
Separated/widowed	1522	21,328	121,031	984,337	1221	11,539	11,654	122,865	15,173	107,878
*Parity*										
Childless	6859	65,867	721,260	7,559,494	5704	58,308	109,760	868,147	105,632	570,147
1 child	2399	28,215	271,704	2,302,232	2330	26,524	16,747	181,907	27,814	187,736
2 + children	4543	92,968	243,758	2,544,750	3772	63,866	19,874	364,698	51,530	621,966
*Employment status*										
Employed	8953	130,485	877,632	7,872,781	7921	100,816	136,975	1,268,466	148,675	1,113,468
Unemployed	1156	12,619	101,295	720,723	358	3233	9406	146,286	4842	27,152
Inactive	3689	43,835	30,993	496,573	1997	25,201	0	0	31,459	234,983
Unknown	3	111	337,505	3,267,249	1530	19,448	0	0	0	4246
*Initial housing tenure*										
Homeowner	5766	122,513	418,170	5,841,273	2336	68,622	–	–	99,963	966,973
Social renter	2225	35,767	189,530	1,405,649	457	5839	–	–	–	–
Private renter	5627	28,077	678,402	2,763,780	8805	73,869	–	–	82,996	401,812
Unknown	183	693	61,323	2,346,624	208	368	–	–	2017	11,064
*Education*										
Low	3897	67,552	268,917	2,595,660	2217	25,741	25,444	304,084	24,955	236,624
Medium	3836	44,193	627,629	5,853,552	6017	81,009	73,036	687,707	119,218	855,081
High	6068	75,305	450,879	3,908,111	3572	41,948	47,901	422,961	40,803	288,144
*Origin group*										
UK natives	9834	126,822	–	–	–	–	–	–	–	–
G1 Europe and West	636	6427	–	–	–	–	–	–	–	–
G1 India	389	3761	–	–	–	–	–	–	–	–
G1 Pakistan	177	3407	–	–	–	–	–	–	–	–
G1 Bangladesh	162	2433	–	–	–	–	–	–	–	–
G1 Caribbean	43	820	–	–	–	–	–	–	–	–
G1 Africa	447	6066	–	–	–	–	–	–	–	–
G2 Europe and West	735	10,571	–	–	–	–	–	–	–	–
G2 India	236	5004	–	–	–	–	–	–	–	–
G2 Pakistan	277	6196	–	–	–	–	–	–	–	–
G2 Bangladesh	167	3886	–	–	–	–	–	–	–	–
G2 Caribbean	237	5470	–	–	–	–	–	–	–	–
G2 Africa	461	6187	–	–	–	–	–	–	–	–
FR natives	–	–	1,141,492	10,379,901	–	–	–	–	–	–
G1 North Africa	–	–	51,229	484,631	–	–	–	–	–	–
G1 Sub–Saharan Africa	–	–	29,611	263,889	–	–	–	–	–	–
G1 South–East Asia	–	–	2884	40,891	–	–	–	–	–	–
G1 Turkey	–	–	8476	104,690	–	–	–	–	–	–
G1 Southern Europe	–	–	13,907	145,750	–	–	–	–	–	–
G1 East Europe	–	–	12,210	111,217	–	–	–	–	–	–
G1 West Europe	–	–	8346	100,770	–	–	–	–	–	–
G2 North Africa	–	–	35,626	318,050	–	–	–	–	–	–
G2 Sub–Saharan Africa	–	–	8508	72,150	–	–	–	–	–	–
G2 South–East Asia	–	–	3499	32,690	–	–	–	–	–	–
G2 Turkey	–	–	3405	36,856	–	–	–	–	–	–
G2 Southern Europe	–	–	18,545	182,739	–	–	–	–	–	–
G2 East Europe	–	–	2349	21,487	–	–	–	–	–	–
G2 West Europe	–	–	7338	58,615	–	–	–	–	–	–
DE natives	–	–	–	–	9720	122,043	–	–	–	–
G1 Polish	–	–	–	–	177	2450	–	–	–	–
G1 Russian-Kazakh	–	–	–	–	267	4331	–	–	–	–
G1 Southern Europe	–	–	–	–	109	1584	–	–	–	–
G1 Turkey	–	–	–	–	84	1749	–	–	–	–
G2 Polish	–	–	–	–	340	3352	–	–	–	–
G2 Russian-Kazakh	–	–	–	–	448	4150	–	–	–	–
G2 Southern Europe	–	–	–	–	279	3715	–	–	–	–
G2 Turkey	–	–	–	–	382	5324	–	–	–	–
CH natives	–	–	–	–	–	–	88,618	864,393	–	–
G1 Ex-Yugoslavia	–	–	–	–	–	–	4936	66,828	–	–
G1 Turkey	–	–	–	–	–	–	1306	15,515	–	–
G1 Southern Europe	–	–	–	–	–	–	7829	93,198	–	–
G1 East Europe	–	–	–	–	–	–	2907	24,718	–	–
G1 West Europe	–	–	–	–	–	–	14,203	124,545	–	–
G2 Ex-Yugoslavia	–	–	–	–	–	–	5129	39,369	–	–
G2 Turkey	–	–	–	–	–	–	1418	10,984	–	–
G2 Southern Europe	–	–	–	–	–	–	12,068	111,894	–	–
G2 East Europe	–	–	–	–	–	–	723	4777	–	–
G2 West Europe	–	–	–	–	–	–	7244	58,531	–	–
SE natives	–	–	–	–	–	–	–	–	157,148	1,199,887
G1 India	–	–	–	–	–	–	–	–	487	2665
G1 North Africa	–	–	–	–	–	–	–	–	769	4961
G1 Middle East	–	–	–	–	–	–	–	–	7369	43,054
G1 Turkey	–	–	–	–	–	–	–	–	918	7389
G1 Poland	–	–	–	–	–	–	–	–	1380	11,200
G1 Ex–Yugoslavia	–	–	–	–	–	–	–	–	2970	25,589
G1Southern Europe	–	–	–	–	–	–	–	–	806	4921
G2 India	–	–	–	–	–	–	–	–	519	3310
G2 North Africa	–	–	–	–	–	–	–	–	610	3558
G2 Middle East	–	–	–	–	–	–	–	–	3584	19,878
G2 Turkey	–	–	–	–	–	–	–	–	1467	10,013
G2 Poland	–	–	–	–	–	–	–	–	1567	9427
G2 Ex–Yugoslavia	–	–	–	–	–	–	–	–	4265	26,082
G2 Southern Europe	–	–	–	–	–	–	–	–	1117	7915
Total	13,801	187,050	1,347,425	12,357,326	11,806	148,698	146,381	1,414,752	184,976	1,379,849

This table presents the number of residential moves and person-years by categories of variables for women separately for each country, e.g., the UK, France, Germany, Switzerland, and Sweden. Education in France is proxied for using the tercile of standard of living

**Table 3:** Number of residential moves to different housing tenure types by origin group. *Source* The UK household longitudinal study (UKHLS), the French permanent demographic sample (PDS), the German socio-economic panel (GSOEP), and a 5% random sample of the Swedish population register, authors’ own calculations. The data analysed is for the period 2010–2019

	UK			France		
	Homeowner	Social renter	Private renter	Homeowner	Social renter	Private renter
Origin Group						
UK natives	4,576	1,555	3,583			
G1 Europe & West	188	83	360			
G1 India	152	21	213			
G1 Pakistan	70	29	77			
G1 Bangladesh	26	63	70			
G1 Caribbean	8	13	21			
G1 Africa	102	162	174			
G2 Europe & West	342	110	276			
G2 India	146	23	63			
G2 Pakistan	138	51	82			
G2 Bangladesh	52	76	35			
G2 Caribbean	73	71	61			
G2 Africa	176	105	174			
FR natives				413,483	121,550	578,788
G1 North Africa				8,055	20,820	21,958
G1 Sub-Saharan Africa				3,974	12,741	12,541
G1 South-East Asia				1,082	514	1,271
G1 Turkey				2,317	2,648	3,449
G1 Southern Europe				3,981	1,512	8,301
G1 East Europe				3,112	2,285	6,692
G1 West Europe				3,297	543	4,408
G2 North Africa				10,133	8,790	15,989
G2 Sub-Saharan Africa				2,120	2,183	3,924
G2 South-East Asia				1,415	409	1,562
G2 Turkey				1,147	882	1,286
G2 Southern Europe				7,115	1,888	9,133
G2 East Europe				835	283	1,129
G2 West Europe				2,622	537	3,917

	Germany			Sweden		
	Homeowner	Social renter	Private renter	Homeowner	Social renter	Private renter
Origin Group						
DE natives	1824	336	7381			
G1 Polish	39	10	128			
G1 Russian-Kazakh	95	25	146			
G1 Southern Europe	25	2	81			
G1 Turkey	26	5	52			
G2 Polish	62	15	255			
G2 Russian-Kazakh	112	27	296			
G2 Southern Europe	56	12	209			
G2 Turkey	97	25	257			
SE natives				88,833	–	66,724
G1 India				169	–	311
G1 North Africa				231	–	512
G1 Middle East				1863	–	5380
G1 Turkey				382	–	521
G1Poland				628	–	722
G1 Ex–Yugoslavia				1307	–	1627
1G Southern Europe				408	–	389
G2 India				288	–	218
G2 North Africa				236	–	361
G2 Middle East				1375	–	2151
G2 Turkey				813	–	639
G2 Poland				782	–	762
G2 Ex-Yugoslavia				2044	–	2182
G2 Southern Europe				604	–	497

Notes: This table presents the number of residential moves to different housing tenure types by origin group for men separately for each country, e.g., the UK, France, Germany, and Sweden. Sweden does not have a ‘social renter’ category

**Table 4:** Hazard ratios (HR) of a residential move, full model and additional model. *Source* The UK household longitudinal study (UKHLS), the French permanent demographic sample (PDS), the German Socio-Economic Panel (GSOEP), the Swiss population register, and a 5% random sample of the Swedish population register, authors’ own calculations. The data analysed is for the period 2010–2019

	Full model		Additional model	
	HR	95% CI	HR	95% CI
Constant	0.05	0.05–0.05	0.04	0.04–0.04
*Sex*				
Male (ref.)	1		1	
Female	1.02	1.02–1.02	1.00	0.99–1.00
*Age*				
15–19 (ref.)	1		1	
20–24	2.70	2.67–2.73	2.17	2.15–2.20
25–29	3.32	3.29–3.36	2.07	2.05–2.10
30–34	2.50	2.47–2.52	1.56	1.54–1.58
35–39	1.76	1.74–1.78	1.17	1.16–1.19
40–49	1.12	1.11–1.13	0.80	0.79–0.81
50–59	0.74	0.73–0.75	0.55	0.54–0.55
*Origin group*				
UK natives (ref.)	1		1	
G1 Europe and West	1.16	1.07–1.25	0.89	0.82–0.96
G1 India	1.38	1.25–1.53	1.14	1.03–1.26
G1 Pakistan	0.72	0.62–0.84	0.69	0.59–0.80
G1 Bangladesh	0.94	0.80–1.09	0.79	0.67–0.92
G1 Caribbean	0.77	0.57–1.04	0.67	0.50–0.91
G1 Africa	0.99	0.90–1.09	0.79	0.72–0.87
G2 Europe & West	0.92	0.86–1.00	0.88	0.82–0.95
G2 India	0.58	0.51–0.66	0.63	0.55–0.72
G2 Pakistan	0.47	0.42–0.53	0.51	0.45–0.57
G2 Bangladesh	0.45	0.39–0.53	0.42	0.36–0.49
G2 Caribbean	0.57	0.50–0.65	0.54	0.47–0.61
G2 Africa	0.80	0.73–0.87	0.74	0.67–0.81
FR natives	1.35	1.33–1.38	1.43	1.40–1.45
G1 North Africa	1.42	1.39–1.45	1.36	1.33–1.39
G1 Sub-Saharan Africa	1.37	1.34–1.40	1.37	1.33–1.40
G1 South-East Asia	1.18	1.13–1.23	1.27	1.22–1.32
G1 Turkey	1.18	1.14–1.21	1.20	1.16–1.23
G1 Southern Europe	1.39	1.35–1.42	1.34	1.30–1.37
G1 East Europe	1.34	1.30–1.38	1.29	1.26–1.33
G1 West Europe	1.24	1.20–1.27	1.33	1.29–1.37
G2 North Africa	1.26	1.24–1.29	1.31	1.28–1.34
G2 Sub-Saharan Africa	1.13	1.10–1.16	1.18	1.14–1.21
G2 South-East Asia	1.20	1.15–1.25	1.33	1.28–1.38
G2 Turkey	1.02	0.99–1.07	1.13	1.08–1.17
G2 Southern Europe	1.23	1.20–1.26	1.33	1.30–1.36
G2 East Europe	1.13	1.08–1.18	1.22	1.17–1.28
G2 West Europe	1.36	1.32–1.41	1.44	1.40–1.48
DE natives	0.99	0.96–1.02	0.72	0.70–0.74
G1 Polish	0.87	0.75–1.01	0.55	0.47–0.64
G1 Russian-Kazakh	0.85	0.75–0.96	0.61	0.54–0.69
G1 Southern Europe	0.93	0.77–1.12	0.56	0.46–0.68
G1 Turkey	0.68	0.55–0.85	0.46	0.37–0.57
G2 Polish	1.19	1.07–1.33	0.81	0.73–0.90
G2 Russian-Kazakh	1.05	0.95–1.15	0.74	0.67–0.81
G2 Southern Europe	0.86	0.76–0.97	0.59	0.53–0.67
G2 Turkey	0.81	0.73–0.90	0.55	0.49–0.61
CH natives	1.58	1.55–1.61	1.50	1.47–1.54
G1 Ex-Yugoslavia	1.67	1.53–1.83	1.24	1.13–1.35
G1 Turkey	1.75	1.62–1.88	1.22	1.13–1.31
G1 Southern Europe	1.99	1.93–2.05	1.28	1.25–1.32
G1 East Europe	1.45	1.36–1.55	1.11	1.04–1.19
G1 West Europe	1.33	1.26–1.40	1.04	0.99–1.10
G2 Ex-Yugoslavia	1.52	1.46–1.59	1.19	1.14–1.24
G2 Turkey	1.65	1.53–1.77	1.42	1.32–1.52
G2 Southern Europe	1.46	1.34–1.60	1.41	1.29–1.54
G2 East Europe	1.67	1.53–1.81	1.19	1.10–1.29
G2 West Europe	1.81	1.74–1.88	1.23	1.19–1.28
SE natives	1.45	1.37–1.53	1.17	1.11–1.24
G1 India	1.59	1.51–1.68	1.36	1.29–1.44
G1 North Africa	1.59	1.53–1.65	1.29	1.25–1.34
G1 Middle East	1.44	1.35–1.53	1.27	1.20–1.35
G1 Turkey	1.34	1.31–1.37	–	–
G1 Poland	1.13	1.09–1.17	–	–
G1 Ex–Yugoslavia	1.22	1.15–1.29	–	–
G1 Southern Europe	1.29	1.25–1.33	–	–
G2 India	1.36	1.30–1.42	–	–
G2 North Africa	1.52	1.48–1.56	–	–
G2 Middle East	1.19	1.15–1.24	–	–
G2 Turkey	1.24	1.17–1.31	–	–
G2 Poland	1.26	1.23–1.30	–	–
G2 Ex–Yugoslavia	1.54	1.43–1.67	–	–
G2 Southern Europe	1.46	1.42–1.50	–	–
*Partnership status*				
Single (ref.)	1		1	
Partnered	0.83	0.83–0.84	1.04	1.04–1.05
Separated	1.65	1.64–1.66	1.15	1.15–1.16
*Education*				
Low (ref.)	1		1	
Medium	1.01	1.00–1.01	0.92	0.92–0.93
High	1.06	1.06–1.07	1.60	1.59–1.61
*Parity*				
Childless (ref.)	1		1	
1 child	1.17	1.16–1.17	1.14	1.14–1.15
2 + children	0.99	0.98–0.99	1.02	1.02–1.03
*Employment status*				
Employed (ref.)	1		1	
Unemployed	1.13	1.12–1.14	1.09	1.08–1.10
Inactive	0.99	0.98–1.00	0.96	0.96–0.97
Unknown	0.96	0.96–0.96	0.94	0.93–0.94
*Origin tenure*				
Homeowner (ref.)	–	–	1	
Social rent	–	–	1.64	1.63–1.65
Private rent	–	–	2.57	2.56–2.58
Unknown	–	–	0.30	0.30–0.30
*Time period*				
2010–2014 (ref.)	–	–	1	
2015–2019	–	–	1.13	1.12–1.13

Notes: Full model—poisson regression for the risk of a residential move—results used in main text. Additional model—as above but including additional variable for origin tenure and time period, and excluding Switzerland

## Footnotes

1 We use the term ethnic minority only when the literature refers to it specifically, it is especially prevalent in UK housing and mobility literature, but we acknowledge that as a group the term includes both immigrants and their descendants.

2 See Appendix A for a detailed description of the data sources.

3 Following Mikolai and Kulu (2018), we considered an alternative definition of a residential change where it is defined as either a change in residence (i.e., move) or a change in tenure type (if there was no residential move). As some tenure changes take place without a residential move. Using this definition of residential change, we conducted sensitivity analysis and found no change in the results.

4 In France both country of birth and nationality at birth are used to define immigrant status. It is commonplace to avoid conflating children of return colonials or expats with immigrant groups. Therefore, those who are born outside of France with French citizenship and those whose parents were born outside of France with French citizenship are excluded. In total this amounts to less than 0.1% of the French sample.

5 In the Swiss data, the children of immigrants are identified based on their parents’ migration status (born in Switzerland or abroad) and their own nationalities. However, if the children of immigrants did not hold a foreign nationality, the information regarding the parents’ origin was lacking. Therefore, these individuals have been excluded from the analysis.

6 Sensitivity analysis where those with missing education in France is used did not alter the results.
